# Roles and mechanisms of CircRNAs in ovarian cancer

**DOI:** 10.3389/fcell.2022.1044897

**Published:** 2022-11-23

**Authors:** Min Liu, Siyu Cao, Ziyi Guo, Zong Wu, Jiao Meng, Yong Wu, Yang Shao, Yanli Li

**Affiliations:** ^1^ Lab for Noncoding RNA and Cancer, School of Life Sciences, Shanghai University, Shanghai, China; ^2^ Department of Gynecologic Oncology, Fudan University Shanghai Cancer Center, Shanghai, China; ^3^ Shanghai Municipal Hospital of Traditional Chinese Medicine, Shanghai University of Traditional Chinese Medicine, Shanghai, China; ^4^ Cancer Institute, Fudan University Shanghai Cancer Center, Shanghai, China; ^5^ Department of Oncology, Shanghai Medical College, Fudan University, Shanghai, China

**Keywords:** ovarian cancer, CircRNAs, biosynthesis, function, drug

## Abstract

Ovarian cancer (OC) is one of the female malignancies with nearly 45% 5-year survival rate. Circular RNAs (circRNAs), a kind of single-stranded non-coding RNAs, are generated from the back-splicing of cellular housekeeping noncoding RNAs and precursor messenger RNAs. Recent studies revealed that circRNAs have different biological function, including sponging miRNAs, encoding micropeptides, regulating stability of cytoplasmic mRNAs, affecting transcription and splicing, *via* interacting with DNA, RNA and proteins. Due to their stability, circRNAs have the potential of acting as biomarkers and treatment targets. In this review, we briefly illustrate the biogenesis mechanism and biological function of circRNAs in OC, and make a perspective of circRNAs drug targeting immune responses and signaling pathways in OC. This article can provide a systematic view into the current situation and future of circRNAs in OC.

## Introduction

### Ovarian cancer

According to the latest cancer statistics, the estimated incidence of ovarian cancer (OC) has reached 19,880, while the predicted number of deaths reached 12,810, ranking fifth among women’s cancer deaths (Siegel et al., 2022). OC could be classified into three broad subgroups: stromal, germ and epithelial cell tumors (Alharbi et al., 2018). 7% of all cases are stromal cell tumors, which develop from the connective tissue surrounding the ovary. The remaining less than 3% are ovarian germ cell tumors that originate from the germ cells of the ovary. Epithelial cell tumors are the most common subtype, accounting for 90% of all cases, and can be classified into type I and type II tumors (Alharbi et al., 2018). Over 75% of OC cases are not found until advanced stages, which makes it difficult to get effective treatment and leads to a high mortality rate (Emmings et al., 2019). Because of the deep location of ovaries in the abdominal cavity, the disease is characterized by vague symptoms, including but not limited to: bloating, dyspepsia, early satiety, altered urinary habits, and generalized pelvic pain and discomfort, which are often overlooked (Stenzel et al., 2019). Although the survival rates for other cancers have improved significantly with medical advances, the 5-year overall survival for OC has barely changed (Lisio et al., 2019). The current clinical standard treatment process includes tumor debulking surgery followed by platinum and paclitaxel chemotherapy. However, chemotherapy resistance has always been one of the biggest obstacles to cancer treatment, including OC. Despite the availability of new chemotherapy regimens such as intraperitoneal delivery and target therapies including poly (ADP-ribose) polymerase (PARP) inhibitors and anti-angiogenics, they have not been effective in improving the 5-year survival rate in advanced OC(Emmings et al., 2019). Therefore, the situation of OC is still serious, and a broader perspective is needed to develop more treatments.

### Biosynthesis of circRNAs

RNA is an important component of genetic information, but only less than 2% of the genome can be translated into proteins (Wang et al., 2019). More than 50,000 non-coding RNAs (ncRNAs) have been found in the last decade and most of them remain unclear (Slack and Chinnaiyan, 2019). Although ncRNAs can’t be translated into proteins, they also are important factors in the regulation of cellular activities. Meanwhile in many diseases, especially cancer, a lot of ncRNAs are significantly dysregulated. ncRNAs can be classified into microRNAs (miRNAs), small nucleolar RNAs (snoRNAs), long ncRNA (lncRNA), and circular RNAs (circRNAs) (Wang et al., 2019). Although ncRNAs mainly serves as biomarkers and therapeutic targets, RNA medicines have been developed and used in the clinic (Adams et al., 2018). More and more basic scientific insights are being used for developing next-generation cancer diagnostics and treatments (Slack and Chinnaiyan, 2019).

As mentioned above, circRNAs are a type of ncRNAs and one of the current research hotspots. CircRNAs are mainly produced by exon cyclization, and while some circRNAs can also be derived from introns, antisense RNAs and 5′ or 3′ untranslated or intergenic genomic regions (Guo et al., 2022). Sanger first identified the structural characteristics of circRNAs, which consist of a single-stranded, highly stable and closed loop structure lacking a 5’cap or 3’poly-A tail (Wei et al., 2021). Unlike conventional forward splicing, circRNAs originate from the same precursor as linear RNA transcriptsto form circular RNAs, which is named as back-splicing (Vromman et al., 2021). Back-splicing creates a covalently closed loop characterized by a non-linear back-splicing junction (BSJ) between the splice donor and upstream splice acceptor (Vromman et al., 2021). Currently, at least four mechanisms are known to modulate circRNAs formation: spliceosome-dependent circularization, intron pairing driven circularization, lariat-driven circularization, and RNA binding proteins-driven (RBPs-driven) circularization (Wu et al., 2020). Due to the covalently structure of circRNAs, they are more stable than linear RNAs and thus resistant to degradation by RNase R. CircRNAs typically exhibit cell-, tissue-, and developmental-stage-specific expression patterns and are highly evolutionarily conserved at the sequence level across species (Cai et al., 2019). Despite the low abundance, the circular structure of circRNAs allows them to be detected in blood, for example, and thus become biomarkers. With the advancement of sequencing technology, more and more circRNAs are coming to the forefront to be known. It has been reported that circRNAs act as important regulatory factors in various diseases such as cancer (Wei et al., 2021), liver diseases (Zeng et al., 2021), skin diseases (Wu et al., 2020), cardiac diseases (Devaux et al., 2017). But much about the mechanism of circRNAs is still unknown and remains to be explored.

Regarding the circRNAs database, circbBase is one of the most commonly used databases. In this database, merged and unified circrna datasets and evidence supporting their expression can be accessed, downloaded and browsed in a genomic context (Glažar et al., 2014). circBase also offers scripts to identify both known and novel circRNA in sequencing data (Glažar et al., 2014). New databases have also emerged in the last few years. For example, CircNet 2.0 (https://ngdc.cncb.ac.cn/databasecommons/database/id/1751) integrates circRNAs from circAtlas and MiOncoCirc, as well as new circRNAs from the Cancer Genome Atlas database, to build high-quality circRNA - miRNA gene regulatory networks (Chen Y. et al., 2022). The databases are good helpers to recognize and study circRNA.

‘‘RNA-Seq,” which means deep sequencing of RNA from biological samples, is a powerful method for discovering and classifying novel alterations in the expression, sequence, and structure of transcriptomes (Salzman et al., 2012). With the great progress in the field of high-throughput RNA sequencing technology, researches of circRNAs has developed rapidly (Shuyuan et al., 2019). When it comes to circRNAs sequencing, there are disadvantages to either short- or long-read deep-sequencing analyses. Since the read lengths available using short-read sequencing data are limited, the accurate internal sequences in circRNAs other than BSJ are often unclear, especially for long circRNAs(Yang et al., 2022). Long-read sequencing methods provide more precise information on circRNAs splicing compared to short-read sequencing, including the identification of intron retention events, microexons, and circRNA-specific exons (Rahimi et al., 2021b). At the same time, many downsides including high cost, biased enrichment of various lengths of circRNAs, and high error rates exist in current long-read sequencing methods (Rahimi et al., 2021a). The latest application of nanopore long-read sequencing (with reads up to 1,000 nt) allows for better identification of circRNAs, offering better annotation by internal selectable splicing (Rahimi et al., 2021a). It is believed that more advanced sequencing methods will be available in the future to make circRNAs sequencing more accurate.

## The perspective of circRNAs in ovarian cancer

Regulated cell death (RCD) can take place in the lack of any exogenous environmental interference and therefore functions as a built-in effector of physiological programs of development or tissue turnover (Galluzzi et al., 2018). These completely physiological forms of RCD are often called programmed cell death (PCD) (Galluzzi et al., 2018). Macroautophagy (hereafter referred to as autophagy) is a lysosome-driven, highly conserved catabolic process that is essential for the maintenance of homeostasis and is also the most common type of PCD (Zhang et al., 2017; Chen J. et al., 2022). Damaged proteins and organelles are engulfed by intracellular phagocytes to produce autophagosomes, which then bind to lysosomes to form autolysosomes (Zhang et al., 2017). The swallowed cargo is degraded by lysosomal hydrolases and the catabolic products are reused or further degraded, allowing the cell to survive in response to external stress (Zhang et al., 2017). There are different characters of autophagy in the types and stages of tumorigenesis. At the early stage, autophagy prevents tumorigenesis and restrains tumor progression (Chen J. et al., 2022). While, once the tumor proceeds to advanced stages and is under stress from the intra- and extracellular environment, autophagy contributes to the survival and proliferation of the primary tumor and increases the aggressiveness of the tumor by promoting metastasis (Chen J. et al., 2022). An increasing number of reports show that ncRNAs, including circRNAs, are associated with autophagy regulation. circRAB11FIP1-induced autophagy speeds up the proliferation and invasion of EOC (Zhang Z. et al., 2021). Whereas, circEEF2 facilitates autophagy, proliferation and invasion of EOC through interaction with miR-6881-3p and ANXA2 (Yong et al., 2020).

Lipophagy is a type of autophagy that specifically engulfs cellular lipid droplets and has been proven to occur in a variety of cells (Zhang S. et al., 2022). And there are two formats of lipophagy: chaperonemediated lipophagy and macrolipophagy (referred to here as lipophagy) (Zhang S. et al., 2022). The mechanism of intracellular lipids regulating autophagy still remains unclear. As with autophagy, little is known about the role of lipophagy in cancer, although analysis of lipophagy receptors has helped to extend the diversity of chemotherapeutic targets (Zhang S. et al., 2022). There is still a gap in research on circRNAs and lipophagy in ovarian cancer, which implies a large emerging targeted therapeutic strategy deserving to be explored.

With the continuous development of targeted therapies, non-apoptotic cell death shows tremendous potential in tumor preventive and therapeutic applications. First of all, ferroptosis presents new perspectives to address the problem of drug resistance. A previous unappreciated mechanism of coupling PARP inhibition to ferroptosis was identified and showed the combination of PARP inhibitors and FINs for the treatment of BRCA-proficient OC (Hong et al., 2021). By targeting SCD1, agrimonolide could serve as a novel apoptosis- and ferroptosis-inducing factor in OC cells. Agrimonolide may be a new drug for the treatment of OC (Liu Y. et al., 2022). Sodium molybdate, a soluble molybdenum compound, can promote ferroptosis in OC cells by increasing labile iron pool (LIP) and decreasing glutathione (GSH) (Mao et al., 2022). Sodium molybdate could also mediate nitric oxide (NO) production, inhibit mitochondrial aconitase activity, ATP production and mitochondrial membrane potential, induce mitochondrial damage, and lead to apoptosis in OC cells. Sodium molybdate could induce ferroptosis and apoptosis in OC cells and is a potential therapeutic agent for OC (Mao et al., 2022). MAP30, a bioactive protein isolated from bitter melon (*Momordica charantia*) seeds, exerts strong anti-cancer and anti-chemotherapy effects on OC cells. MAP30 exhibits a synergistic effect on cisplatin-induced cytotoxicity in OC cells, while MAP30 also induces an increase in intracellular Ca^2+^ ion concentration, triggering ROS-mediated cancer cell death through apoptosis and ferroptosis (Chan et al., 2020). There are few studies on circRNA affecting iron death in OC, but there are a number of relevant studies in other cancers. Exosomes and circRNA_101,093 are critical for desensitization of lung adenocarcinoma cells to ferroptosis (Zhang X. et al., 2022). Both circRNA as well as ferroptosis are recent research hotspots, and hopefully the collision of the two will open new horizons for OC treatments.

Pyrogenesis is mediated by the gasdermin protein, which forms pore and promotes immune cell activation and infiltration by releasing pro-inflammatory cytokines and immunogenic substances after cell rupture (Loveless et al., 2021). Pyroptosis can rapidly lead to tumor degeneration on the one hand and promote the development of the tumor microenvironment on the other hand (Loveless et al., 2021). As a critical regulator of multiple cancers, silencing of HOTTIP leads to suppression of cell proliferation and NLRP1 inflammasome-mediated pyroptosis (Tan et al., 2021). Excessive pyroptosis may lead to an overwhelming and persistent inflammatory response, involved in inflammatory diseases, and may be a novel strategy for tumor eradication through the induction of pyroptosis cells and potent activation of antitumor immunity (Rao et al., 2022). Nobiletin is a promising new anti-OC drug candidate derived from citrus fruits. Nobiletin decreases mitochondrial membrane potential, induces ROS generation and autophagy, and promotes gasdermin D-/gasdermin E-mediated pyroptosis (Zhang R. et al., 2020). Bexarotene, also a possible new therapeutic agent for OC, activates caspase-4 and GSDME to induce pyroptosis (Kobayashi et al., 2022). 2-(anaphthoyl)ethyltrimethylammonium iodide (α-NETA) induces EOC cell pyroptosis *via* the gesdermin-d (GSDMD)/caspase-4 pathway (Qiao et al., 2019). Compared with other cell death modalities, pyroptosis is more niche and mysterious. Overall, there are few studies on circRNAs and pyroptosis in OC, but pyroptosis is also a strong potential stock in future cancer therapy.

## Regulatory function of circRNAs in ovarian cancer

CircRNAs own diverse biological functions. The unique life cycle of circRNAs, biological origin, conformation and binding partners, and long-term stability all facilitate its cellular regulatory potential (Yang et al., 2022).

### Regulation of transcription

First of all, circRNAs could affect RNA polymerase Ⅱ (RNA Pol Ⅱ) transcription. So far there are two experimentally proven mechanisms to explain this phenomenon, and here’s the first one. CircRNAs can bind strongly to their cognate DNA locus with forming RNA: DNA hybrids, or R-loops, which can affect DNA transcription, repair and replication (Conn et al., 2017; García-Muse and Aguilera, 2019). While some circRNAs, such as *ci-ankrd52*, contain high GC% and tend to form R-loops for RNase H1 cleavage, this process seems to encourage transcriptional elongation of Pol Ⅱ at the ciRNA-producing loci (Li X. et al., 2021). EIciRNAs enhance the expression of their parental genes in cis, performing transcriptional control by means of specific RNA-RNA interactions between U1 snRNAs and EIciRNAs (Li et al., 2015).

The second one is co-activating transcription factors (TFs). For example, binding of cia-MAF to the MAFF promoter recruits the TIP60 complex to the MAFF promoter and ultimately boosts MAFF expression in Liver tumor-initiating cells (Chen Z. et al., 2021). In mesenchymal tumors, circPOK regulates pro-proliferative and pro-angiogenic factors by acting as a coactivator of ILF2/3 upon ILF2/3 binding to the proximal promoter of II6 (Guarnerio et al., 2019). In hepatocellular carcinoma, circIPO11 recruits TOP1 to the GLI1 promoter, triggering its transcription and leading to the activation of Hedgehog signaling (Gu et al., 2021).

### Scaffold or sponge for protein

Certain circRNAs have been demonstrated to bind significantly to proteins, but do not change the properties of the protein, because these circRNAs may provide a platform or scaffold for proteins. cDOPEY2 serves as a protein scaffold to potentiate the interaction between the E3 ligase TRIM25 and cytoplasmic polyadenylation element binding protein (CPEB4), thereby promoting the ubiquitination and degradation of CPEB4 in esophageal squamous cell carcinoma (Liu et al., 2021). In osteoarthritis tissues, circPDE4B is used as a scaffold to promote the association between RIC8A and MID1 by promoting RIC8A degradation *via* proteasomal degradation (Shen et al., 2021).

CircMbl was first found to sponge proteins. The existence of functional MBL binding sites in the flanking intron sequences allows MBL to increase circMbl production. The sequences in both introns are, however, necessary for the circRNAs circularization process, indicating that MBL induces circularization by linking the two flanking introns (Ashwal-Fluss et al., 2014). Similarly, cia-cGAS binds to the DNA sensor cGAS in the nucleus and blocks its synthase activity, hence protecting dormant LT-HSCs from cGAS-mediated depletion in LT-HSCs (Xia et al., 2018). Meanwhile *cia-cGAS* possessed a stronger binding affinity to cGAS than self-DNA, resulting in the inhibition of cGAS-mediated production of type I IFNs (Xia et al., 2018).

Furthermore, some circRNAs could regulate cellular activity by forming complex called circRNPs. CircACC1 in colorectal cancer tissues forms a ternary complex with the regulatory β and γ subunits to stabilize and promote the enzymatic activity of AMPK holoenzyme (Li et al., 2019). Inhibition of cell cycle progression by ectopic expression of circFoxo3 is achieved by binding to the cell cycle protein -dependent kinase 2 (also known as cytokinin kinase 2 or CDK2) and cell cycle protein-dependent kinase inhibitor 1 (or p21), thereby forming a ternary complex (Du et al., 2016). circRNA SCAR is mediated by PGC-1a, binds to ATP5B, and shuts down mPTP through blocking CypD-mPTP interactions in mitochondria (Zhao Q. et al., 2020). The interaction of YBX1 (Y-box binding protein 1) with Nedd4l (E3 ubiquitin ligase) was enhanced by circNfix, and the expression of cyclin A2 and cyclin B1 was inhibited by ubiquitination-induced degradation of Ybx1 (Huang et al., 2019).

### Sponge for miRNAs

CircRNAs, which are localized in the cytoplasm with signaifcant stability, have been shown in many studies to act as competitive endogenous RNA (ceRNAs) to regulate miRNA and thus genes or signaling pathways. circMAP3K5 could isolate miR-223p and thus inhibit TET2 expression (Shi et al., 2022), and then TET2 mediates vascular SMC differentiation. CircACTN4 promotes intrahepatic cholangiocarcinoma proliferation and metastasis by acting as a sponge for miR-424-5p and by interacting with YBX1 to transcriptionally activate FZD7, which is upregulated in intrahepatic cholangiocarcinoma expression (Chen Q. et al., 2022). CircLMO7 affects the WNT2/β-Catenin pathway as a miR-30a-3p sponge and promotes gastric cancer cell proliferation, migration and invasion (Cao et al., 2021). circRNF144B served as a sponge for miR-342-3p and inhibited miR-342-3p-induced lysine demethylase 2 A (FBXL11) mRNA degradation, resulting in elevated FBXL11 protein levels (Song et al., 2022). Increased FBXL11 promoted Beclin-1 ubiquitination and degradation, thereby inhibiting autophagy (Song et al., 2022). Similar regulation is reflected in other cancers. Ectopic expression of circ-LNLM can promote colorectal cancer cell invasion and induce liver metastasis by directly combining with AKT (Tang et al., 2021). Phosphorylation of AKT (T308/S473) was initiated owing to the blockage of the ubiquitination site of Lys in the 0-52aa peptide of circ-LNLM (Tang et al., 2021).

CDR1as (antisense transcript of the cerebellar degenerationrelated protein 1), also called ciRS-7, is found to modulate miR-7 stability or transport in neurons, while miR-671 regulates CDR1as levels (Piwecka et al., 2017) And in CDR1as-deficient mice, both miR-7 and miR-671 were mis-regulated, demonstrating a physiological interaction between CDR1as and miRNAs in brain function (Piwecka et al., 2017). Regarding the sponge interaction of miRNAs and circRNAs, the mechanism may be more sophisticated than what we previously perceived. The second highest molecule that interacts with miR-7 is lncRNA Cyrano, which has an arguably perfect binding site (Piwecka et al., 2017). Cyrano combines miR-7 and contributes to miR-7 destruction by inducing its 3′-terminal tail and trimming, which in turn allows CDR1as to accumulate in the brain (Kleaveland et al., 2018). Further, in Cyrano-deficient mice, increased miR-7 led to degradation of CDR1as in the cerebellum, partly because miR-671 enhanced the slicing of CDR1as.

### Interaction with mRNAs

There is also a certain possibility that circRNAs binds directly to mRNA thereby affecting mRNA expression. CircZNF609 directly interacts with a few mRNAs and enhances their stability and translation by supporting the recruitment of the RNA-binding protein ELAVL1 (Rossi et al., 2022). The interaction site with CKAP5 mRNA overlaps the back-splicing sequence, thus enhancing the translation of CKAP5, regulating microtubule function and maintaining cell cycle progression in cancer cells (Rossi et al., 2022). In orbitofrontal cortex (OFC) and stem cell-derived neurons in culture from subjects with psychiatric disorders, a negative correlation was found between circHomer1 and the relative levels of HOMER1B mRNA isoforms (Hafez et al., 2022). It was demonstrated that circHomer1 can combine the 3′ UTR of Homer1b mRNA and that mature circHomer1 and Homer1b mutually repress each other’s synaptic expression, by using *in vivo* circHomer1-and Homer1b-specific knockdown in mouse OFC (Hafez et al., 2022).

### Translation into protein

The field of circRNAs research is rapidly heating up because it has been discovered to have a translatable function. If a circRNA could possess a translatable open reading frame (ORF) containing a start codon, then translation would theoretically be possible. The exact mechanism of cap-independent translation of circRNAs remains obscure and has individual specificity. Internal ribosome entry sites (IRESs) have been a strong contender for the circRNA translation mechanism. IRESs were the first regulatory elements of mRNA translation identified in viruses, but under extreme conditions of stress such as hypoxia and viral invasion, eukaryotes could also perform translation *via* IRESs (Godet et al., 2019). In eukaryotes, the N^6^-methyladenosine (m6A) is the most enriched internal modification of RNA, and although some circRNAs do not have natural IRES, a single m6A site is adequate to trigger translation (Yang et al., 2017). Meanwhile by recruiting initiation factor eIF4G2 and m6A reader YTHDF3, m6A modifications can also drive the translation of circRNAs (Yang et al., 2017). This mechanism is another cap-independent conversion mechanism called MIRES, which remains unclear (Meyer et al., 2015). It was shown that methyltransferase and demethylase could enhance and inhibit translation, respectively (Shi et al., 2020). Yet the latest report demonstrates that translation of circRNAs may be easier and more common than we thought. Multiple short IRES-like elements were found, suggesting that circRNAs translation may not be reliant on IRES (Fan et al., 2022). The rolling translation product of circRNAs was first identified and has been verified using plasmid expression system (Fan et al., 2022).

**FIGURE 1:**
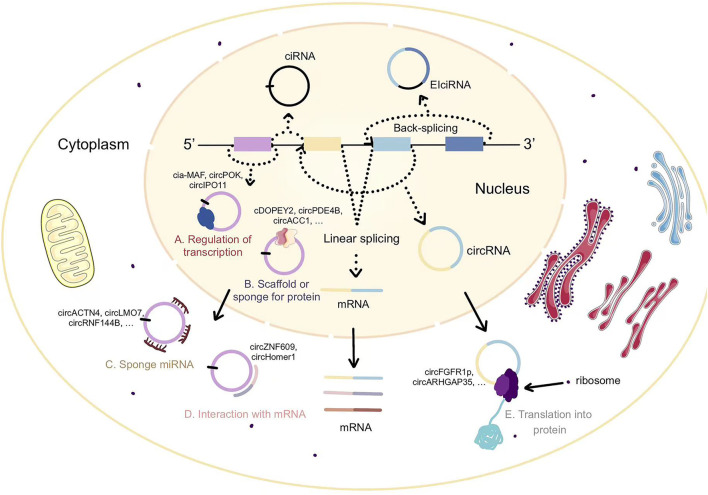
The biosynthesis and function of circRNAs.

There have been many studies on the characteristics of circRNA-encoded proteins. Circ-ZNF609 with two start codons may translate into two proteins in a splicing-dependent and cap-independent manner (Legnini et al., 2017). The UTR element of circ-ZNF609 from the termination to the start codons can drive IRES-dependent translation, but is only generated by splicing events, indicating that factors loaded on the transcript at splicing may play a key role in ribosome recognition and translation initiation (Legnini et al., 2017). The circRNA-encoded protein circFGFR1p acts as a negative regulator of FGFR1 through a dominant-negative mechanism, inhibiting cell growth under stressful conditions (Chen C. K. et al., 2021). The translation initiation of circFGFR1p is likewise by IRES (Chen C. K. et al., 2021). UTRs of ribo-circRNAs (cUTRs) permit cap-independent translation, starvation and FOXO may regulate translation of circMbl isoforms (Pamudurti et al., 2017). In hepatocellular carcinoma, circARHGAP35 consists of a 3,867 nt ORF with an m6A-modified start codon and encodes a truncated protein consisting of four FF structural domains that lack the Rho GAP domain (Li Y. et al., 2021). Furthermore, circARHGAP35 has an opposite expression and function to linear ARHGAP35. CircARHGAP35 protein facilitates cancer cell progression through interaction with TFII-I protein in the nucleus (Li Y. et al., 2021). SMO-193a.a. is encoded by circular SMO (circ-SMO) and is required for SMO activation induced by sonic hedgehog (Shh) by interacting with SMO, reinforcing SMO cholesterol modification, and releasing SMO from the suppression of patched transmembrane receptors (Wu et al., 2021). Circ-SMO/SMO-193a.a. is under positive regulation by FUS, the direct transcriptional target of Gli1, and Shh/Gli1/FUS/SMO-193a.a. forms a positive feedback loop in glioblastoma, maintaining the activation of Hedgehog signaling (Wu et al., 2021). A never-mentioned secretory E-cadherin protein variant (C-E-Cad) is an additional activation mechanism of EGFR signaling in glioblastoma through multiple-round open reading frame translation of circular E-cadherin (circ-E-Cad) (Gao et al., 2021). C-E-Cad maintains the tumorigenicity of glioma stem cells by binding to the EGFR CR2 domain *via* a unique 14-amino-cid carboxy terminus, which activates EGFR independently of EGF (Gao et al., 2021). Nevertheless, the experimental data also showed that the abundance of circRNA-encoded proteins was relatively low. There are two possible reasons for the low abundance of circRNAs translation products, one is due to the low abundance of circRNAs itself, and the other is due to the rapid degradation of circRNAs translation products (Fan et al., 2022) (Figure 1).

## The function of circRNAs in ovarian cancer

### Biomarkers

In OC, similarly, there are many studies on circRNAs. First of all, circRNAs could be biomarkers of OC. CircHIPK3 expression was more abundant in the sequencing, and silencing CircHIPK3 facilitated the proliferation, migration and invasion of OC cells and normal ovarian epithelial cells and suppressed apoptosis (Teng et al., 2019). In addition, increased circHIPK3 expression is correlated with lymph node infiltration, FIGO (International Federation of Gynecology and Obstetrics) stage, patients’ DFS and OS, so circHIPK3 may be a new biomarker for EOC prognosis (Liu et al., 2018). CircEXOC6B (Ning et al., 2018) and circ N4BP2L2 (Ning et al., 2018) are also biomarkers for OC. CircRNA1656 was expressed down-regulated in high-grade ovarian serous carcinoma (HGSOC) tissues and OC cell lines, evidently correlated with HGSOC FIGO stage, and likewise has the potential to be a new tumor marker for HGSOC (Gao et al., 2019). The expression of hsa_circ_0003972 and hsa_circ_0007288 (circCOMBO) was down-regulated in plasma, tissues and cell lines of OC patients, which possessed diagnostic value and might be used as new circulating biomarkers for OC diagnosis (Ge et al., 2022). Among them, high expression levels of hsa_circ_0007288 in plasma and OC tissues were related to lower lymph node metastatic potential in OC (Ge et al., 2022).

Circ_0078607 is derived from SLC22A3 and inhibits the proliferation and invasion of OC cells by sponge oncogenic miR-518a-5p inducing Fas expression and promoting apoptosis (Zhang N. et al., 2020). The expression of circLARP4 was correlated significantly with FIGO staging and lymph node metastases, which is also potential biomarkers of OC prognosis (Zou et al., 2018). Inhibition of VPS13C--hsa-circ-001567 significantly promoted apoptosis and suppressed proliferation of SKOV3 and OV-1063 cells (Bao et al., 2019). After knockdown of VPS13C-hsa-circ-001567, the cell cycle was stopped in G1 phase, the proportion of S1 phase cells decreased, the invasive ability of SKOV3 and OV-1063 cells was reduced, and the expression levels of E-cadherin and N-cadherin were altered (Bao et al., 2019). Moreover, the knockdown of VPS13C-hsa-circ001567 gene significantly reduced the tumorigenicity of OC cells (Bao et al., 2019).

Exosomes, also one of the hot spots of research, are one of the ways in which circRNA is used as a biomarker. Circulating exosome circFoxp1 can serve as a biomarker for EOC and a potential therapeutic target (Luo and Gui, 2020).

### Involvement in drug resistance

Circ-LPAR3 was significantly upregulated in cisplatin (DDP) -resistant OC tissues and cells, miR-634 could interact with circ-LPAR3, and PDK1 was targeted by miR-634 (Liu et al., 2022a). Thus circ-LPAR3 may be involved in DDP resistance in facilitating OC *via* the miR-634/PDK1 axis (Liu et al., 2022a). The overexpression of circITGB6 promotes M2 macrophage-dependent CDDP resistance, while circITGB6 knockdown works just the opposite (Li et al., 2022). Mechanistically circITGB6 interacts directly with IGF2BP2 and FGF9 mRNA to form circITGB6/IGF2BP2/FGF9 RNA-protein ternary complexes in the cytoplasm, thus stabilizing FGF9 mRNA and inducing TAMs to polarize toward the M2 phenotype (Li et al., 2022).

The development of novel drugs is also being accelerated today, and curcumin has a potential therapeutic effect on OC (Sun and Fang, 2021). And curcumin inhibited ovarian cancer cell proliferation by regulating the circPLEKHM3/miR-320a/SMG1 axis, and promoted apoptosis (Sun and Fang, 2021).

CircFoxp1 induces DDP resistance in EOC cells and positively regulates the expression of CCAAT enhancer binding protein gamma (CEBPG) and formin like 3 (FMNL3) through miR-22 and miR-150-3p (Luo and Gui, 2020). Propofol, as a common intravenous anesthetic, has antitumor effect in a variety of cancers, including OC (Lu et al., 2021). Propofol inhibits OC cell proliferation, cell cycle, migration and invasion through circVPS13C/miR-145/MEK/ERK signaling pathway and induces apoptosis in ovarian cancer cells *in vitro* (Lu et al., 2021). Isoproterenol inhibits glycolysis by mediating the cyclic RNA-zinc finger RNA binding protein (ZFR)/microRNA (miR)-212-5p/superoxide dismutase 2 (SOD2) axis. In addition, Propofol inhibits cancer cell progression by regulating the above axis. By mediating the circular RNA-zinc finger RNA-binding protein (ZFR)/microRNA (miR)-212-5p/superoxide dismutase 2 (SOD2) axis, propanol inhibits glycolysis (Qu et al., 2022). Furthermore, propanol inhibits cancer cell progression by regulating this certain axis (Qu et al., 2022).

### Regulation of proliferation and metabolism

CircRNAs influences the proliferation and metabolism of OC cells as well as various phenotypes. circRNA-UBAP2 was upregulated in OC tissues and cell lines and targeted miR-382-5p to downregulate its expression, and PRPF8 was a target gene of miR-382-5p (Xu et al., 2020). The circRNA-UBAP2/miR-382-5p/PRPF8 axis affects OC through a competitive endogenous RNA (ceRNA) mechanism (Xu et al., 2020). CircMTO1, downregulated in OC tissues and cell lines, can sponge miR-182-5p to support KLF15 expression, ultimately leading to inhibition of OC progression (Wang et al., 2020). The circE2F2 expression was remarkably upregulated in OC tissues and cell lines, and high circE2F2 expression was associated with poorer survival (Zhang M. et al., 2021). Mechanically, circE2F2 stabilizes E2F2 mRNA by binding to HuR protein and promotes OC cell proliferation, metastasis and glucose metabolism (Zhang M. et al., 2021).

The expression of circular RNA-ITCH was downregulated, whereas the expression of lncRNA HULC was upregulated (Yan et al., 2020). CircRNA-ITCH was significantly and negatively correlated with HULC, and circRNA-ITCH may suppress the proliferation of OC through down-regulating HULC (Yan et al., 2020). Meanwhile circ-ITCH inhibits EOC cell proliferation and promotes apoptosis through sponging of miR-10a (Luo et al., 2018). In addition, the circ-ITCH-miR-145-RASA1 axis exerted the same inhibition effect *in vitro* and *in vivo* (Hu et al., 2018). Furthermore, hsa_circ_0051240 serves as a sponge for miR-637, which directly targets KLK4 mRNA in OC cells and promotes OC proliferation, migration and invasion (Zhang M. et al., 2019). And circ-PVT1 promotes EOC cell proliferation but inhibits apoptosis by sponging miR-149 (Sun et al., 2020).

Circ-FAM53B is associated with clinical severity and poor prognosis in patients with OC (Sun et al., 2019). Together with circ-FAM53B may be a ceRNA that competes for the sponge of miR-646 and miR-647 to upregulate the expression of VAMP2 and MDM2 at the post-transcriptional level, thereby mediating the cellular behavior of OC cells (Sun et al., 2019). Circ-MYLK contributes to OC malignant progression possibly by regulating microRNA-652 and is significantly associated with poor prognosis (Zhao Y. et al., 2020). Circ-ABCB10, which promotes EOC cell proliferation and reduces apoptosis, negatively regulates miR-1271, miR-1252 and miR-203, and is associated with advanced clinicopathological features and poor survival of EOC (Chen et al., 2019). Circ_0000745 isolates miR-3187-3p, prevents its inhibitory effect on ERBB4, and ERBB4 promotes phosphorylation of PI3K/AKT signaling pathway (Wang et al., 2021). In addition, circ_0000745 is upregulated by IGF2BP2, which promotes OC cell invasiveness and stemness through the above axis (Wang et al., 2021).

Specifically, circPLEKHM3 exerts oncogenic effect in OC cells by targeting the miR-9/BRCA1/DNAJB6/KLF4/AKT1 axis, and affects EMT as well as taxol resistance (Zhang L. et al., 2019). Circ_0007841, as a ceRNA for miR-151-3p promotes the expression of MEX3C, and recovery of MEX3C levels recovers the proliferation, migration, and invasive ability of OC cells (Huang et al., 2021). CircWHSC1 mediates upregulation of downstream targets MUC1 and hTERT expression *via* sponge-mediated miR-145 and miR-1182 (Zong et al., 2019). Together with the fact that exosomal circWHSC1 can translocate to peritoneal mesothelial cells and promote peritoneal dissemination (Zong et al., 2019).

Interestingly, circRNAs could bind to its source gene and thus perform biological function. CircCRIM1 binds to miR-145-5p as a competitive endogenous RNA (ceRNA) for CRIM1 (Du et al., 2021). And circCRIM1 combines with miR-383-5p to increase ZEB2 expression in OC (Du et al., 2021). CircCRIM1 and CRIM1 both promote the progression of OC (Du et al., 2021).

### Regulation of epithelial-mesenchymal transition

Epithelial-mesenchymal transition (EMT) is likewise affected by circRNAs. High expression of hsa_circ_0013958 was closely associated with FIGO stage and lymph node metastasis in patients (Pei et al., 2020). Knockdown of hsa_circ_0013958 inhibited the proliferation, migration and invasion of ovarian cancer cells, but increased the rate of apoptosis (Pei et al., 2020). Also, the expression levels of epithelial-mesenchymal transition-related protein and apoptosis-related protein were altered (Pei et al., 2020).

CiRS-7, a competitive endogenous RNA for miR-641, promotes OC cell growth and metastasis by regulating ZEB1 and MDM2-mediated EMT (Zhang F. et al., 2020). High expression of ciRS-7 was associated with TNM stage, lymph node metastasis status and overall survival in OC patients (Zhang F. et al., 2020). Plus circ_0000554 promotes OC cell growth, invasion and EMT by sponging miR-567 (Wang et al., 2022). Similarly, circFGFR3 induces EMT in OC cells *via* the miR-29a-3p/E2F1 axis thereby promoting OC development (Zhou et al., 2020).

### Regulation of signaling pathways

The regulation of signaling pathways also includes circRNAs. Hsa_circ_0009910 is associated with poor prognosis in OC patients and regulates NF-κB and Notch pathways through the management of miR-145 (Li et al., 2020). Hsa_circ_0009910’s significant synergistic effect with miR-145 may be used in the treatment of OC (Li et al., 2020). Also, the expression of circ9119 was significantly reduced in OC tissues and cell lines, which could regulate the proliferation and apoptosis of OC cells by targeting the phosphatase and tensin homologue (PTEN) 3′ UTR as miR-21 sponge, thus affecting the PTEN-Akt pathway (Gong et al., 2020). CircFBXO7, a bone fide tumor suppressor, acts as a ceRNA for miR-96-5p for regulating the expression of MTSS1 (Wu et al., 2022). Thus, downregulation of MTSS1 causes excessive accumulation of β-catenin and increased phosphorylation of GSK3β, resulting in translocation of β-catenin to the nucleus, which activates the Wnt/β-catenin signaling pathway and subsequently promotes ovarian cancer progression (Wu et al., 2022).

Circ-PGAM1 directly interacts with miR-542-3p and negatively feeds back to each other (Zhang C. et al., 2020). CDC5L is a direct target of miR-542-3p, an oncogene in OC (Zhang C. et al., 2020). In addition, CDC5L protein directly binds to the PEAK1 promoter and promotes its transcription (Zhang C. et al., 2020). The overexpression of PEAK1 activates the ERK1/2 and JAK2 signaling pathways and promotes the malignant progression of OC.

The function of circRNAs in OC could also be related to autophagy. Silencing of circRAB11FIP1 suppressed autophagic flux in SKOV3 cells, while circRAB11FIP1 overexpression activated autophagic flux in A2780 cells, suggesting that circRAB11FIP1 is associated with autophagy (Zhang Z. et al., 2021). Experimental results showed that the binding of circRAB11FIP1 to desmocollin 1 facilitated its interaction with ATG101 (Zhang Z. et al., 2021). Eventually circRAB11FIP1-induced autophagy accelerates the proliferation and invasion of EOC (Zhang Z. et al., 2021). CircRAB11FIP1 regulates ATG7 and ATG14 by sponging miR-129 and mediates ATG5 and ATG7 mRNA expression levels through m6A modification (Zhang Z. et al., 2021). In addition to these results circRAB11FIP1 directly binds to mRNAs of fat mass and obesity-associated protein and contributes to its expression (Zhang Z. et al., 2021) (Table 1).

**TABLE 1:** The list of circRNAs in OC.

Gene name	Regulation	Tissue	Cell lines	Functions	Mechanism	Target genes	Source
circHIPK3	↑	n = 21; n = 69	A2780, SKOV3, IOSE80; A2780, HO8910, SKOV3, CAOV3, HOEC	facilitated the proliferation, migration and invasion of ovarian cancer cells and suppressed apoptosis; correlated with lymph node infiltration, FIGO stage, patients’ DFS and OS	miRNA sponge; a novel biomarker for EOC prognosis	miR-10a-5p is the most abundant miRNA related	(Liu et al., 2018; Teng et al., 2019)
circEXOC6B	↓	n = 54			potential diagnostic biomarkers		Ning et al. (2018)
circ N4BP2L2	↓	n = 54			potential diagnostic biomarkers		Ning et al. (2018)
circRNA1656	↓	n = 3	SKOV3, HO8910, A2780, OVCAR-3, human ovarian epithelial cells	significantly associated with HGSOC FIGO stage	a novel tumor marker		Gao et al. (2019)
hsa_circ_0003972	↓	n = 41/15	A2780, SKOV3, IOSE386	possessed diagnostic value, may function as ceRNA	new circulating biomarkers		Ge et al. (2022)
hsa_circ_0007288 (circCOMBO)	↓	n = 41/15	A2780, SKOV3, IOSE386	possessed diagnostic value, may function as ceRNA	new circulating biomarkers		Ge et al. (2022)
circ_0078607	↓	n = 18	SKOV3, A2780	inhibits the proliferation and invasion of OC cells and promoting apoptosis	sponge miR-518a-5p inducing Fas expression	miR-518a-5p	Zhang et al. (2020c)
circLARP4	↓	n = 78		correlated significantly with FIGO staging and lymph node metastases	a potential biomarker of OC prognosis		Zou et al. (2018)
VPS13C-hsa-circ-001567	↑	n = 20	IOSE80, ES-2, SKOV3, Caov-3, and OV-1063	VPS13C-has-circ-001567 plays a role in the development of OC	a prognostic marker and therapeutic target		Bao et al. (2019)
circFoxp1	↑	n = 194	COC1, OVCAR3, SKOV3, SKOV3/DDP, IOSE-80	circFoxp1 can serve as a biomarker for EOC and a potential therapeutic target	circulating exosome		Luo and Gui, (2020)
circ-LPAR3	↑		SKOV3, A2780	enhances theDDP resistance of OC	miR-634/PDK1 axis	miR-634	Liu et al. (2022a)
circITGB6	↑	n = 119	OVCAR3, SKOV3, TOV21G, TOV112D, CAOV3, A2780	promotes M2 macrophage-dependent CDDP resistance	circITGB6/IGF2BP2/FGF9 RNA-protein ternary complexes	IGF2BP2 and FGF9 mRNA	Li et al. (2022)
circPLEKHM3	↓	n = 35	SKOV3, A2780, 293 T	curcumin restrained proliferation and facilitated apoptosis	circPLEKHM3/miR-320a/SMG1 axis	miR-320a	Sun and Fang, (2021)
circFoxp1	↑	n = 194	COC1, OVCAR3, SKOV3, SKOV3/DDP, IOSE-80	induces DDP resistance in EOC cells and positively regulates the expression of CEBPG and FMNL3	miR-22 and miR-150-3p	miR-22 and miR-150-3p	Luo and Gui, (2020)
circVPS13C	↑	n = 40	IOSE-80, A2780, SKOV3, 293 T	propofol inhibits OC cell proliferation, cell cycle, migration and invasion and induces apoptosis	circVPS13C/miR-145/MEK/ERK	miR-145	Lu et al. (2021)
circ-ZFR			A2780	propanol inhibits glycolysis and cancer cell progression	circ-ZFR/miR-212-5p/SOD2 axis	miR-212-5p	Qu et al. (2022)
circRNA-UBAP2	↑	n = 20	IOSE80, SKOV3, OVCAR-3, ES-2, A2780	promote OC cell proliferation and inhibit apoptosis	circRNA-UBAP2/miR-382-5p/PRPF8 axis	miR-382-5p	Xu et al. (2020)
circMTO1	↓	n = 48	SKOV3, OVCAR3, IOSE80	inhibition of OC progression	miR-182-5p/KLF15 axis	miR-182-5p	Wang et al. (2020)
circE2F2	↑	n = 70	OVCAR-3, SKOV3, A2780, CAOV3, IGROV1, ES2, HOSEpiC	promotes OC cell proliferation, metastasis and glucose metabolism	stabilizes E2F2 mRNA by binding to HuR protein	E2F2 mRNA	Zhang et al. (2021a)
circular RNA-ITCH	↓	n = 75	UWB1.289 + BRCA1, UWB1.289	suppress the proliferation of OC	down-regulating HULC		Yan et al. (2020)
Circ-ITCH	↓		SKOV3, A-2780, OVCAR-3, HO-8910, IOSE80	inhibits EOC cell proliferation and promotes apoptosis	sponging of miR-10a	miR-10a	Luo et al. (2018)
Circ-ITCH	↓	n = 20	SK-OV-3, Caov-3	inhibits the malignant progression of OC	circ-ITCH-miR-145-RASA1 axis	miR-145	Hu et al. (2018)
hsa_circ_0051240	↑	n = 33	CAOV-3, SKOV-3, OVCAR-3, H8910, HOSE	promotes OC proliferation, migration and invasion	hsa_circ_0051240/miR-637/KLK4 axis	miR-637	Zhang et al. (2019b)
Circ-PVT1	↑		CAOV3, SKOV3, OVCAR3, HOSEpiC	promotes EOC cell proliferation but inhibits apoptosis	sponge miR-149	miR-149	Sun et al. (2020)
circ-FAM53B	↑	n = 54	HO8910, SKOV3, OVCAR3, A2780, iOSE80	mediates the cellular behavior of OC cells	circ-FAM53B/miRNA-646/VAMP2 and circ-FAM53B/miRNA-647/MDM2	miRNA-646, miRNA-647	Sun et al. (2019)
circRNA_MYLK	↑	n = 46	SKOV3, OVCAR3, PEO1, 3AO, A2780, CAOV3, HOSEPiCs	contributes to OC malignant progression possibly	regulating microRNA-652	microRNA-652	Zhao et al. (2020b)
Circ-ABCB10	↑	n = 103	OVCAR3, UWB1.289, SKOV3, CAOV3, IOSE80	promotes EOC cell proliferation and reduces apoptosis	negatively regulates miR-1271, miR-1252 and miR-203	miR-1271, miR-1252 and miR-203	Chen et al. (2019)
circ_0000745	↑	n = 50	IOSE-80, CoC1, ES-2, SW626, SK-OV-3	promotes OC cell invasiveness and stemness	miR-3187-3p/ERBB4/PI3K/AKT axis	miR-3187-3p	Wang et al. (2021)
circPLEKHM3	↓	n = 10	A2780, OV90, MDAH2274	exerts oncogenic effects and affects EMT as well as Taxol resistance	miR-9/BRCA1/DNAJB6/KLF4/AKT1 axis	miR-9	Zhang et al. (2019a)
circ_0007841	↑	n = 43	IOSE80, SKOV3, OVCAR3	promotes the expression of MEX3C	circ_0007841/miR-151-3p/MEX3C axis	miR-151-3p	Huang et al. (2021)
circCRIM1	↑	n = 154	OVCAR3, CAOV3	circCRIM1 and CRIM1 both promote the progression of OC	miR-383-5p/ZEB2 axis	miR-145-5p; miR-383-5p	Du et al. (2021)
circWHSC1	↑	n = 92	CAOV3, OVCAR3	translocate to peritoneal mesothelial cells and promote peritoneal dissemination	regulating MUC1 and hTERT through sponging miR-145 and miR-1182	miR-145 and miR-1182	Zong et al. (2019)
hsa_circ_0013958	↑	n = 90	A2780, OVCAR-3, HOSEpiC	may be involved in the development of OC by affecting epithelial-mesenchymal transition and apoptotic signaling pathways			Pei et al. (2020)
ciRS-7	↑	n = 40	SKOV3, A2780, OV 2008, IGROV1, ES-2, HOSE	promotes OC cell growth and metastasis	ciRS-7/miR-641/ZEB1 or ciRS-7/miR-641/MDM2 axis	miR-641	Zhang et al. (2020b)
circ_0000554	↑	n = 40	HG-SOC, SKOV-3, HO8910, HO8910PM, FTE187	promotes OC cell growth, invasion and EMT	sponge miR-567	miR-567	Wang et al. (2022)
circFGFR3	↑	n = 35	OSE, SKOV3, A2780, OV 2008, IGROV1	induces EMT thereby promoting OC development	circFGFR3/miR-29a-3p/E2F1 axis	miR-29a-3p	Zhou et al. (2020)
Hsa_circ_0009910	↑	n = 50	SKOV3	regulates NF-κB and notch pathways	induction of proliferative and motile phenotypes by inhibition of miR-145	miR-145	Li et al. (2020)
circ9119	↓	n = 50	SKOV-3, HO-8910, A2780, ES-2, CAOV3, OVCAR3, FTE187	regulate proliferation and apoptosis	sponge miR-21, targeting PTEN 3′ untranslated region	miR-21	Gong et al. (2020)
circFBXO7	↓	n = 53	A2780, MDAH2774, OV90, SKOV3, HEK 293 T	promotes OC progression	circFBXO7/miR-96-5p/MTSS1 axis	miR-96-5p	Wu et al. (2022)
circ-PGAM1	↑	n = 45	CAOV3, SKOV3, OVCAR3, ES-2, 293T	circPAGM1 silencing coupled with miR-542-3p overexpression exerted maximal anti-cancer effects	circ-PGAM1/miR-542-3p/CDC5L/PEAK1	miR-542-3p	Zhang et al. (2020a)
circRAB11FIP1	↑	n = 100	A2789, SKOV3	associated with autophagy speeds up the proliferation and invasion of EOC	regulates ATG7 and ATG14 by sponging miR-129	miR-129	Zhang et al. (2021b)

“↑” represents upregulation, and “↓” represents downregulation.

## Conclusion and perspectives

Cancer cells are thought to have a lot of cunning because they flexibly adapt molecular and cellular mechanisms to survive under the stress of drug interference, leading to the evolution and regeneration of more aggressive or metastatic phenotypes that are no longer sensitive to treatment (Yung et al., 2022). And to date, the molecular mechanisms of cancer drug resistance are still not fully understood. Therefore circRNAs, and even ncRNAs, still has a long way to go from the laboratory to the clinic. Besides, circRNAs is expected to be a future target for OC treatment as well as reversal of drug resistance. The use of circRNAs in clinical applications is currently uncommon and mostly remains a rosy vision of future therapeutic targets. Thus, more studies are needed to better understand the role of the circRNAs in chemotherapy resistance.

Since COVID-19 became a global pandemic disease, mRNA vaccines have made a big splash. Possessing both translatable and stable characteristics, circRNA holds great potential for the production of therapeutic peptides/proteins as peptide/protein replacement treatments and vaccines (Liu et al., 2022b). Current research on improving the *in vitro* synthesis method of circRNAs is in full swing since the first exogenous circRNAs was shown to translate protein in eukaryotic cells (Wesselhoeft et al., 2018). Orna Therapeutics, the first company in the world to specialize in the development of new therapies using circRNAs, was founded in 2019. Orna has by now demonstrated that its circRNAs can drive protein expression to levels that are therapeutically valuable in animal models of human disease (Garber, 2022). Orna Therapeutics has currently announced four lines of development: *in situ* CAR-T cell therapy, Duchenne Muscular Dystrophy (DMD) therapies, COVID-19 vaccines, and a pipeline of undisclosed information. Recent research has also developed new rapid synthesis methods for circRNAs that increase circRNAs protein yields by hundreds of times (Chen R. et al., 2022). How to accurately and effectively deliver circRNAs to targeted cells for tumor therapy also remains an important issue needing to be addressed. As an emerging star molecule, circRNAs has its own special advantages and offers new prospects and directions for cancer and further diseases.
